# Radical Cystectomy is the best choice for most patients with muscle-invasive bladder cancer? Opinion: No

**DOI:** 10.1590/S1677-5538.IBJU.2017.02.04

**Published:** 2017

**Authors:** Timur Mitin

**Affiliations:** 1 Department of Radiation Medicine, Oregon Health and Science University, Portland, Oregon

**Keywords:** Urinary Bladder Neoplasms, Cystectomy, Chemoradiotherapy, Adjuvant, Neoadjuvant Therapy

Progress is a hallmark of human civilization. Over the past centuries, it became easier, faster and safer to travel from point A to point B, and the choices of transportation are now numerous, making it at times challenging for travelers to make their selection and forcing them to buy guidebooks, research travel websites and sometimes even contact the travel agents. Perhaps sailing was once the only way of crossing the ocean, but with advent of aviation it is no longer the case. Similarly, upfront radical surgeries were once patients’ only hope for cure - radical mastectomy for patients with breast cancer, amputation for patients with extremity sarcoma, laryngectomy for patients with laryngeal cancer, radical prostatectomy for patients with prostate cancer, abdominoperineal resection for patients with anal carcinoma. However, over the past 40 years the field of oncology has embraced organ preservation, often, but not always, through randomized clinical trials showing equivalent outcomes with smaller surgeries and adjuvant radiation therapy, or replacement of upfront surgery with definitive chemoradiation therapy, reserving organ extirpation for salvage in case of local recurrences. The management of muscle-invasive bladder cancer (MIBC) is no different (1).

Stating that one treatment – such as radical cystectomy – is the best choice for most patients with a particular disease – such as muscle-invasive bladder cancer – is no different from advertising a particular mode of transportation. One treatment never fits all, and the best choice of treatment modality for an individual patient starts with a multi-disciplinary evaluation and inclusion of patients and their caregivers in the decision process. An open discussion of all issues related to radical cystectomy (RC) and reconstruction options on one hand, and tri-modality bladder preservation therapy with chemoradiation (TMT) on the other hand allows for the ultimate informed consent and highest patient satisfaction. Most discussions of bladder preservation for MIBC are centered on two themes – efficacy and toxicity.

**Survival of patients who undergo cystectomy or tri-modality bladder preservation**

A UK randomized trial “SPARE” for patients with MIBC closed in 2007 due to poor accrual, in part because patients did not want to be randomized to the surgical arm. Based on 45 randomized patients, the trial showed no difference in overall survival, a trend towards decreased post-treatment grade 3 and 4 toxicity in the bladder preservation arm, and a salvage cystectomy rate of 18% for patients with local recurrence following TMT (2). An Egyptian randomized trial of 160 patients with MIBC, which included a high percentage of patients with squamous cell carcinoma of the bladder – a histology more common in Egypt than other countries -- showed no difference in overall survival between upfront radical cystectomy vs TMT (3). A high rate of salvage cystectomies (30%) due to local recurrences were due to low radiation dose of 46 Gy, a dose that is not used in current European and North American TMT practices. Similar 5- and 10-year survival rates from large published cystectomy and bladder preservation treatment series (4) made the National Comprehensive Cancer Network (NCCN) Bladder Cancer Guidelines committee in 2013 accept tri-modality bladder preservation therapy as an alternative treatment option for patients fit to undergo radical cystectomy.

In 2015 Italian physicians have conducted a meta-analysis and compared the overall survival outcomes in over 3,000 patients treated with TMT on 29 published studies to outcomes in over 10,000 patients treated with RC on 30 clinical series. The worse 5-year overall survival rates were associated with undergoing RC (5). What could explain the observed inferiority of RC? Radical cystectomy has a significant risk of post-operative mortality, which increases as patients age. A large SEER database analysis of over 10,000 patients treated in the US with RC between 1984 and 2004 showed that at 90 days mortality is 1% for patients younger than 60 years of age, 6% between ages 69 and 83 and 14% for patients over age 89 (6). If post-operative mortality could be reduced by limiting cystectomy to young and healthy patients, one could hypothesize that cystectomy outcomes would be in line, or superior, to outcomes with tri-modality bladder preservation treatment.

It is important to consider TMT as an attempt to preserve the native bladder without jeopardizing the ultimate outcomes for patients with MIBC - survival. The vast experience in Europe and North America indicates that over 70% of carefully selected patients with MIBC achieve durable local control and avoid the need for salvage cystectomy. Patients who have undergone bladder preservation therapy must be followed closely by their urologists with frequent cystoscopic evaluations, so that an early local recurrence in 30% of these patients could be effectively treated with salvage cystectomy. The reconstructive options may be limited for these patients, due to presence of irradiated bowel, which may be unacceptable for a continent reservoir or a neobladder creation. However, peri-operative morbidity and mortality rates in almost 100 patients who underwent salvage cystectomy at Massachusetts General Hospital (7) were remarkably similar to rates for immediate cystectomies without radiation as reported by other centers specializing in treatment of bladder cancer.

**Quality of life of patients who undergo cystectomy or tri-modality bladder preservation**

The most common myth pertains to the overwhelming radiation-induced side-effects and poor quality of life for patients who opt to keep their bladders. Among 157 patients who underwent TMT for MIBC on national clinical protocols with a median follow-up of 5.4 years, only 7% experienced late grade 3 genitourinary or gastrointestinal toxicities (8). There were no late grade 4 toxicities and no treatment-related deaths. No cystectomies were performed due to treatment-related toxicity in any of these patients. 200 patients were followed with a median follow-up of 6 years and were asked to undergo urodynamics study (9) and fill out quality of life questionnaires (10). Seventy-five percent of patients had normal functioning bladders by UDS. Reduced bladder compliance was seen in 22% of patients, however only a third of these patients experienced distressing bladder symptoms. Bowel symptoms occurred in 22% of patients and caused distress in 14% of patients. Thirty-six percent of men reported normal erections and another 18% less firm erections, but sufficient for intercourse. Of note, the median age of male patients in this study at the time of the questionnaire was 68 years. The rate of erectile dysfunction after RC, on the contrary, is almost 100%.

A recent retrospective study of 173 patients who underwent TMT at Massachusetts General Hospital [64], or RP at University of North Carolina [109] on multivariate analysis revealed association between TMT treatment and better general quality of life, better bowel function, and better sexual function, while urinary symptoms were similar between the two group of patients (11).

**Clinical situations when RC may be desirable for patients with MIBC**

A multi-disciplinary evaluation of patients and discussion of treatment options may reveal that a particular patient may be a suboptimal candidate for TMT. History of prior pelvic irradiation for other malignancies may prevent additional dose of radiation that would be adequate for bladder tumor eradication. Poor baseline bladder function due to tumor and/or previous intravesical therapies, such as BCG or mitomycin, may indicate that upfront RC would be expected to lead to better quality of life in these particular clinical scenario. For large or multi-focal tumors that cannot be grossly removed by maximal transurethral resection of bladder tumor (TURBT), or in the setting of hydronephrosis, the local control with chemoradiation therapy may be lower than 70%. Finally, patients who adamantly desire neobladder may decide to pursue upfront RC, in order to avoid a situation when neobladder may not be possible in the setting of local recurrence after TMT.

## CONCLUSIONS

Multi-disciplinary evaluation of patients with MIBC and discussion of all treatment options is key to best outcomes and patient satisfaction (12), similarly to other malignancies (13). Ultimately, overall survival for patients with MIBC – showed to be in 40-50% range at 5 years in large published series of RC and TMT (4) - is dictated not so much by the choice of local therapy – radical cystectomy or TMT with chemoradiation therapy – but by the high risk of systemic disease progression and comorbid medical conditions that are so prevalent in patients afflicted with bladder cancer. Based on growing evidence in the literature, TMT is associated with similar or better outcomes to cystectomy for patients with MIBC. Quality of life in these patients is excellent. Similarly to other solid malignancies, local recurrence rate is significant, necessitating a thorough and frequent cystoscopic follow-up for patients after completion of tri-modality bladder preservation. Thirty percent of patients will require salvage cystectomy for local disease recurrence, usually identified within the first three years, and the morbidity and mortality from salvage cystectomy is no different than from upfront radical cystectomy.

Is sailing from France to England the best option for most travelers? A “Yes” answer is not substantiated by safety and satisfaction results from studies of randomly assigning travelers to various transportation modes. A “Yes” answer may not fit the needs of particular travelers exploring their options. A “Yes” answer is clearly biased towards financial support of the ferryboat industry.
